# Liquid Metal Embrittlement of Advanced High Strength Steel: Experiments and Damage Modeling

**DOI:** 10.3390/ma14185451

**Published:** 2021-09-21

**Authors:** Konstantin Manuel Prabitz, Mohammad Zhian Asadzadeh, Marlies Pichler, Thomas Antretter, Coline Beal, Holger Schubert, Benjamin Hilpert, Martin Gruber, Robert Sierlinger, Werner Ecker

**Affiliations:** 1 Materials Center Leoben Forschung GmbH, Roseggerstraße 12, 8700 Leoben, Austria; mohammad.asadzadeh@mcl.at (M.Z.A.); marlies.pichler@mcl.at (M.P.); werner.ecker@mcl.at (W.E.); 2 Institute of Mechanics, Montanuniversitaet Leoben, Franz Josef-Straße 18, 8700 Leoben, Austria; thomas.antretter@unileoben.ac.at; 3 Institute of Materials Science, Joining and Forming, Graz University of Technology, Kopernikusgasse 24/I, 8010 Graz, Austria; coline.beal@gmail.com; 4 Mercedes-Benz AG, 71059 Sindelfingen, Germany; holger.schubert@daimler.com (H.S.); benjamin.hilpert@daimler.com (B.H.); 5 voestalpine Stahl GmbH, voestalpine-Straße 3, 4020 Linz, Austria; martin.gruber@voestalpine.com (M.G.); robert.sierlinger@voestalpine.com (R.S.)

**Keywords:** liquid metal embrittlement, advanced high strength steel, resistance spot welding, damage modeling, finite element modeling, machine learning, symbolic regression, genetic programming

## Abstract

In the automotive industry, corrosion protected galvanized advanced high strength steels with high ductility (AHSS-HD) gain importance due to their good formability and their lightweight potential. Unfortunately, under specific thermomechanical loading conditions such as during resistance spot welding galvanized, AHSS-HD sheets tend to show liquid metal embrittlement (LME). LME is an intergranular decohesion phenomenon leading to a drastic loss of ductility of up to 95%. The occurrence of LME for a given galvanized material mainly depends on thermal and mechanical loading. These influences are investigated for a dual phase steel with an ultimate tensile strength of 1200 MPa, a fracture strain of 14% and high ductility (DP1200HD) by means of systematic isothermal hot tensile testing on a Gleeble^®^ 3800 thermomechanical simulator. Based on the experimental findings, a machine learning procedure using symbolic regression is applied to calibrate an LME damage model that accounts for the governing quantities of temperature, plastic strain and strain rate. The finite element (FE) implementation of the damage model is validated based on the local damage distribution in the hot tensile tested samples and in an exemplary 2-sheet resistance spot weld. The developed LME damage model predicts the local position and the local intensity of liquid metal induced cracking in both cases very well.

## 1. Introduction

Modern advanced high strength steels (AHSS) combine high tensile strength with ductility and are therefore highly in demand in the automotive industry. To fulfil emission norms in conventionally-driven vehicles and to compensate the heavy weight of batteries in e-mobility, it is essential to achieve lightweight design. Facing these demands, AHSS are excellent candidate materials for the use in the car body in white at comparatively low costs while enhanced safety can be guaranteed. On top of that, the high ductility AHSS grade (AHSS-HD) offers exceptional formability. To protect the car body from corrosion, the steel sheets are galvanized, i.e., covered with a thin zinc layer that acts as cathodic protection [1]. The main method of joining these sheets is resistance spot welding (RSW) due to its reliability, potential for automation and cost efficiency. Per vehicle, up to 5000 of such spot welds are placed.

During RSW, the galvanized AHSS-HD sheets exhibit high temperatures far above the melting point of zinc (419 °C) and liquid metal embrittlement (LME) might occur under specific conditions. LME basically describes brittle cracking of an otherwise ductile metal. During LME, zinc penetrates at the steel grain boundaries at low strains, so grain cohesion decreases, which can lead to damage or even complete structural failure. The propagation of the crack is limited by the supply of zinc to the crack tip and the local loading conditions [2,3]. The initial cracks form due to wetting of the susceptible base metal and lead to rapid crack progression along the grain boundaries after reaching a critical crack length. Stress-assisted grain penetration is seen as the occurring penetration mechanism while low-energy grain boundaries suppress LME [4]. Razmpoosh et al. [5] described that ordered grain boundaries show less embrittlement than high-misorientation-angle grain boundaries. Besides the strength level of the AHSS-HD and the type of coating, the main influences to LME are the material pairing, temperature and the level of plastic strain [6]. The occurrence of tensile plastic strain combined with temperatures above 700 °C is used in the literature as a rule of thumb to estimate the risk of crack formation [7,8,9,10]. Other influences such as loading speed [11], microstructure and alloying metals are also reported to have an impact on LME susceptibility [12,13]. Different testing methods such as hot dip testing of cups with residual stresses [14], modified welding process parameters and welding tests with applied external tensile load [15,16], fracture mechanics testing [17] or hot tensile testing [18,19,20] have been proposed to investigate the influence of LME to steel. Among those, uniaxial hot tensile tests are most widely employed because of their ease of use, repeatability and reliability. The thermal and mechanical loading can be varied within a wide range of parameters relevant for LME. Resistance heating allows high heating rates and strain rates in the same order of magnitude as expected during RSW. The results allow comparability to similar tensile tests for other base material-coating pairs. The literature reports research results for different steel grades such as TRIP (transformation-induced plasticity) with a yield strength of 1100 MPa [21] and TWIP (twinning-induced plasticity) [22] steels. By contrast, this work focuses on a dual phase (DP) steel with high ductility and an ultimate tensile strength (UTS) of 1200 MPa. To use the full potential of this steel grade, it is necessary to reliably determine LME critical loading conditions. Implementing this knowledge into a finite element (FE) code allows to predict LME and to ultimately optimize the RSW process. This will serve as the basis for suggesting LME prevention measures, e.g., modification of the electrode tip geometry [23] or the welding conditions [24].

This work presents a quantitatively reliable experimental testing method for analyzing the LME susceptibility of DP1200HD sheets. The experimental results are used to develop an LME damage model based on a well-structured and repeatable machine learning workflow using symbolic regression. The damage model is implemented into a FE model whose predictions are validated against crack networks in tensile testing samples and on RSW spot welds. 

## 2. Materials and Methods

### 2.1. Experimental

**Figure 1:**
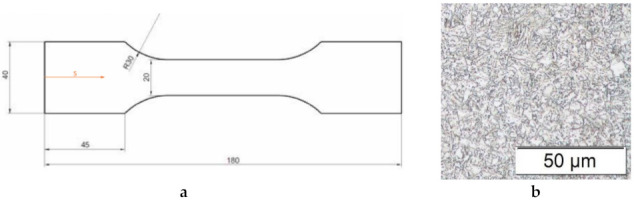
(**a**) Geometry of the hot tensile testing specimen and (**b**) the initial microstructure.

The investigated DP steel offers a UTS of 1200 MPa and provides high ductility with a fracture strain of 14% (DP1200HD). For comparison reasons, bare, i.e., uncoated (UC) and electrolytically galvanized (EG) flat tensile specimens with a zinc layer of 7 μm thickness on both faces were machined from a 1.6 mm thick sheet material. The long axis of the samples and, hence, the loading direction, is oriented in the direction of rolling. The total length of the dog bone-shaped specimen was 180 mm with a strain gauge length of 45.28 mm and a width of 20 mm as shown in Figure 1a. Figure 1b shows the initial microstructure.

The investigated DP1200HD steel showed an initial microstructure consisting of martensite and ferrite, which both transform to pure austenite above the A_C3_ temperature (870 °C) while A_C1_ lies at 775 °C. Table 1 shows the chemical composition of the steel.

A Gleeble^®^ 3800 was used to simulate realistic welding conditions. Similar to the actual RSW process, conductive materials were subjected to resistance heating at the Gleeble^®^ thermomechanical testing facility [8]. The length measurements were carried out by multiple means: (i) monitoring the stroke along the longitudinal path, (ii) an L-gauge measuring the longitudinal elongation as well as (iii) a C-gauge for the transversal contraction. Type K thermocouples were applied for temperature control and measurement. They exhibit an accuracy of ± 2.2 °C for temperatures between 0 °C up to 293 °C and ± 0.75% from 293 °C up to 1260 °C according to ASTM E230. A control thermocouple was placed in the center while two others were placed 11 mm and 22 mm away from the center along the tensile axis for measuring the thermal gradient. The samples were heated to testing temperature and subsequently subjected to tensile load until fracture. The fractured samples were then cooled on air. The experiments were performed for a temperature range from 25 °C up to 1200 °C for the UC condition and from 500 °C up to 1000 °C for EG with a constant heating rate of 300 °C/s and strain rates ranging from 0.01 s^−1^ up to 10.0 s^−1^. Altogether, 105 tests were performed and the onset of LME was determined up to 12.5 °C accurately. The standard copper clamping jaws were substituted with steel jaws, while additional graphite foil and tantalum sheets were added between jaws and sample for providing a homogeneous temperature distribution. For each set of parameters, at least two repeated tests were carried out. The raw data was analyzed with Origin 2018 as well as a specific Python script that computes the ratio of the deformation energies of EG over UC. It automatically uses the matching data sets of the hot tensile tests for an accurate and precise data evaluation without adding human errors. Finally, the cut open samples were digitized and further evaluated.

In addition, RSW tests with a broad range of welding parameters were carried out by means of a C-type welding gun and an X-type welding gun equipped with a Nimak pedestal welder and a Matuschek controller. The welding parameters in the presented example used for model validation were as follows: electrode force = 4.5 kN, welding current = 6.9 kA, welding time = 380 ms and holding time = 300 ms. The electrode type was chosen to be a F1–16-20–6 [25], manufactured from a copper alloy according to [26].

### 2.2. Modeling

In this work, we develop an LME damage model based on experimental data and a machine learning procedure to be used as an input to the FE solver. It is usually preferable to deal with analytical models represented by mathematical equations in a closed form. Hence, symbolic regression is applied for the machine learning task.

### 2.3. Symbolic Regression

**Figure 2:**
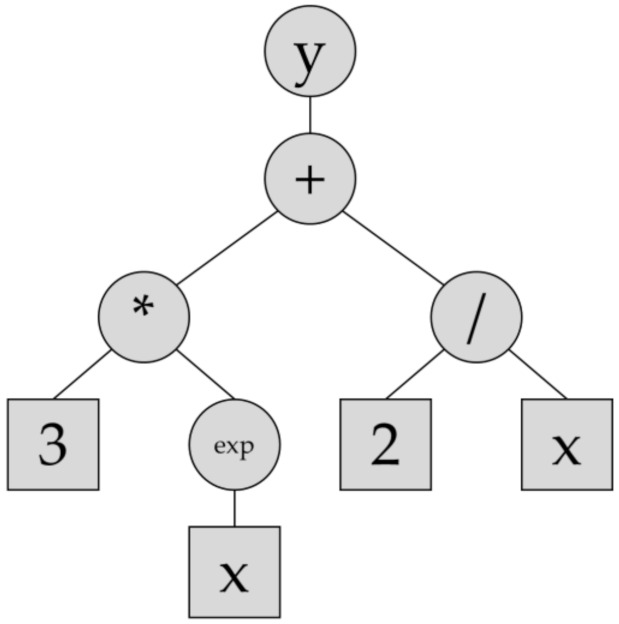
An example of an expression tree representing the function y = 3e^x^ + 2/x.

Symbolic regression (SR) is a machine learning approach capable of extracting non-trivial patterns from input data. The user defines all allowed functions and the software applies them to find an equation that matches the input data. In contrast to other machine learning approaches such as Artificial Neural Networks (ANN), SR finds an explicit mathematical formula which describes a target variable as a function of input variables. Therefore, in SR, in contrast to classical regression, not only the model parameters but also the model structure is optimized. The search space is infinite and is comprised of mathematical operations, functions, constants and variables. Therefore, SR is usually applied with genetic programming (GP) to evolve from an initial set of solutions (this includes model structures and model parameters), such that an optimum solution is found according to a given fit metric. In order to reduce the dimension of search space, the mathematical building blocks shall be provided to the algorithm. By applying genetic algorithms such as crossover and mutation, GP creates new solutions or populations, similar to natural evolution, which better satisfy the fit function. A solution in GP is represented as an expression tree. For example, a solution such as $y=3e^{x}+2/x$ is represented as it is shown in Figure 2.

A tree is comprised of instruction and leaf nodes. Instruction nodes embed operations and functions while leaf nodes contain constants and variables. It is clear that more complex trees are readily constructed by combining simpler trees as branches. Each tree is constrained by depth (i.e., the number of layers) and length (i.e., the number of nodes). For more detail, the interested reader is referred to the original work by John Koza in which he presented SR during his GP developments [27].

We use the open-source software, HeuristicLab, version 3.3.15, for SR modeling [28]. The following settings and parameters are set for the optimizer. The ‘Offspring selection genetic algorithm’ is selected for the evolution of the initial solutions, with population size = 10,000, mutation probability = 5%, maximum tree length = 30, maximum tree depth = 10, number of elites = 2, maximum selection pressure = 100, generation = 100, crossover operator = MultiSymbolicData-AnalysisExpressionCrossover, mutator = MultiSymbolicExpressionTreeManipulator, selector = proportional and analyzer = Multianalyzer. The functions and operations are limited to {+, *, -, /, EXP()} applicable on the instruction nodes while the leaf nodes are fed with numeric constants and input variables of the training dataset. The optimization stops when the maximum number of generations is reached or the selection pressure reaches the limit of 100. Inspecting the optimization history a posteriori confirms that this criterion suffices to achieve convergence. The optimization procedure was repeated ten times starting from random initial configurations in order to avoid getting trapped in local minima. The best model is selected as the one with the lowest root mean squared error.

After analysis of the experimental data (see Experimental Results), the influencing factors of the damage model are identified. These characteristic quantities, i.e., temperature, plastic strain and strain rate behave not necessarily proportional to the fracture strain. Therefore, again, SR is employed to find the mathematical expression for the fracture strain $\varepsilon_{f}$ as a function of strain rate $\dot{\varepsilon }$ and temperature T. This damage model is to be used in the FE models of the RSW process.

### 2.4. Finite Element Method

The LME damage model was implemented in the commercial software package Abaqus 2019 [29] by means of a FORTRAN 77-based user subroutine of the type UVARM. In order to verify the implementation, single element tests were carried out representing the hot tensile tests at the material point level. For the sake of validation, 3-dimensional models capturing the full geometrical and multi-physical details of the hot tensile tests were developed. The thermal and mechanical boundary conditions were set in accordance to the temperature measurements and the loading prevalent at the fracture point for all different thermo-mechanical tests conducted with the Gleeble^®^. As element type, the electrical-, thermal- and mechanically-coupled hexahedral elements labeled Q3D8 were used. A mesh convergence study proofed that a structured mesh with an element size of 0.1 mm sufficiently discretize the sample. The necessary material models were taken from a previous publication on validated FE modeling of the RSW process by the authors [30]. This RSW model for the welding parameters described in the Experimental section was also applied as a second means of validation of the damage model in this work. The RSW model covers the evolution of the electrical, thermal, mechanical and metallurgical fields during the complete spot welding process. Phase transformations such as from the base material to austenite and subsequently to the melt during heating and all relevant transformations during cooling were considered. Details on the model are provided in Ref. [30].

## 3. Results and Discussion

### 3.1. Experimental Results

**Figure 3:**
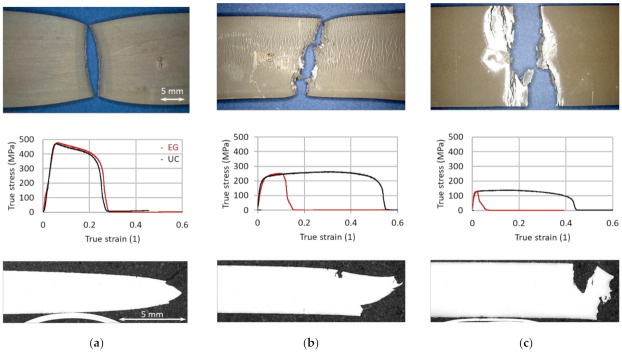
Macro documentation of the fracture surface (top view), stress-strain curves and micrographs of the axial cuts for EG samples with a strain rate of 0.1 (1/s) at (**a**) 600 °C, (**b**) 725 °C and (**c**) 900 °C.

Figure 3 shows macroscopic images of three EG test samples and their according stress-strain curves for UC (black) and EG (red) material state for a strain rate of 0.1 s^−1^. Micrographs for axial center cuts through the fracture surface were machined and are shown on the bottom of Figure 3. For UC samples, typical ductile necking occurs which is also observed for EG at 600 °C in Figure 3a. Figure 3b shows reduced necking for the EG steel and surface cracks neighboring the main crack. Both phenomena are clear signs of LME at 725 °C. At 900 °C, these signs become even more pronounced as shown in Figure 3c. The tendencies of the stress-strain curves corroborate the conclusions drawn from examining the micrographs. 

**Figure 4:**
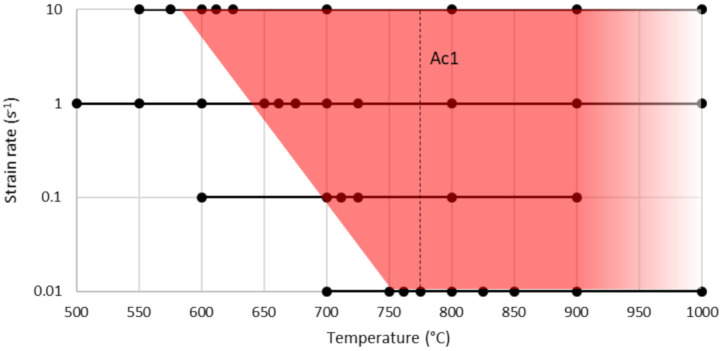
Testing schedule and visible LME in red for a variation of strain rates and temperatures. The dots mark the exact testing temperature.

For the investigated specimens, LME only occurs as anticipated in EG samples but not in UC samples. As the name liquid metal embrittlement implies, the zinc coating has to be liquid (419.53 907 °C) [31]. The defined minimum temperature to obtain LME is additionally dependent on the different material pairings and strain rates. Following the scheme exemplarily shown in Figure 3, all available samples were examined. The results are summarized in Figure 4. The black dots represent the temperatures at which hot tensile tests were done. The red coloured area in Figure 4 marks the parameter space in which LME occurred. Jung et al. [7] stated that the onset of LME is somehow promoted by the transformation to the austenitic phase. By contrast, in our study, the onset of LME for the investigated DP1200HD is for high strain rates clearly below the A_C1_ temperature of 775 °C. Hence, LME does not depend on the occurrence of austenite but also happens in the martensitic and ferritic microstructure present at temperatures as low as 575 °C. Similar findings are reported in the very recent paper of Bhattacharya et al. [32].

In Figure 4, the inverse behavior of the temperature critical for LME becomes evident. When decreasing the strain rate, the critical temperature for triggering LME becomes higher. This phenomenon can be explained by the formation of intermetallic iron zinc phases (e.g., α-Fe(Zn)) that have a higher melting point than pure zinc and do postpone the formation of LME. Low strain rates combined with high temperatures are the reason for the formation of these intermetallic phases [14]. The intermetallic phases form at the interface of Fe-Zn and suppress the contact between steel and liquid zinc while acting as a protection layer of the base steel [33]. High-magnitude tensile stresses can cause fracture of the α-Fe(Zn) layer and continue in the steel sheet, while liquid zinc can penetrate the cracks and initiate LME.

**Figure 5:**
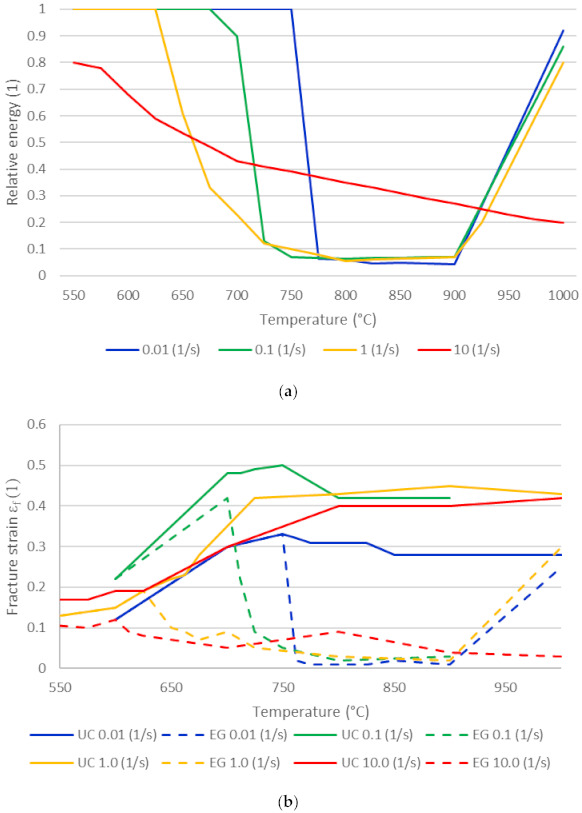
(**a**) Relative fracture energy between EG/UC and (**b**) fracture strain for all tested strain rates of EG/UC steel.

In the literature, the ductility trough, which is typical for LME, indicates the loss of ductility as a function of temperature for different strain rates [34,35,36]. In this study, the most pronounced LME can be found at tests with low strain rates and high temperatures, which indicates that liquid zinc shows a limited wetting speed at the crack tip. Due to slow straining, more time is available for zinc to move along the crack tip. Another aspect is that due to low strain rates (0.01 s^−1^), the homogeneous temperature range becomes narrow in comparison to the tests with higher strain rates. Additionally, intermetallic phases form between zinc and steel and a reduction of LME is found. Note that the relevant strain rates for the later application in RSW ranges roughly from 0.1 s^−1^ up to 10 s^−1^. Figure 5a represents the energy ratio that is defined as deformation energy of the EG steel divided by the deformation energy of the UC steel [37], while Figure 5b shows the fracture strain. It can be seen that the width of the ductility trough becomes larger with increasing strain rate while the magnitude of embrittlement stays maximal above a specific temperature except for 10 s^−1^ where the magnitude of embrittlement constantly increases with increasing temperature. The shape of the ductility trough at the highest strain rates can either be explained by a limited zinc supply speed and, therefore, shorter cracks as well as by crack shielding mechanisms due to the presence of many short cracks. This is both indicated by the reduction of elongation, which is not as pronounced as for lower strain rates, as well as by micrographs showing numerous cracks. A partial embrittlement and some residual ductility occur.

For strain rates ranging from 0.01 s^−1^ up to 1.0 s^−1^, a significant drop of the energy ratio occurs at a specific temperature, while for 10 s^−1^, it is not as pronounced. Annealing plays a major role in this case. The lower the strain rate, the more time is available and more zinc is able to penetrate at the grain boundaries and cause a more pronounced rupture behavior, but also more intermetallic phases are formed that delay the start of LME. Above the vaporization temperature of zinc, a recovery of ductility is found for strain rates up to 1.0 s^−1^ [38]; see Figure 5a. A damage model solely based on the strain energy was found to be inappropriate for non-isothermal loading. For the ductile and semi ductile fracture that is driven by plastic deformation, the plastic fracture strain plotted in Figure 5b is analyzed to serve as the basis for the damage model outlined below.

### 3.2. Damage Model

#### 3.2.1. Symbolic Regression

**Figure 6:**
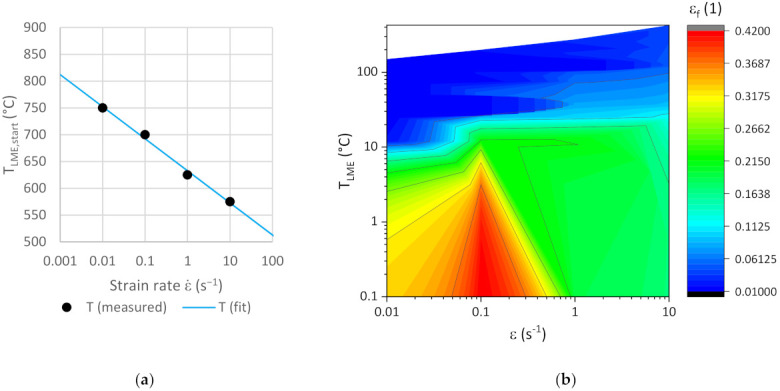
(**a**) Minimum temperature for LME depending on the strain rate and (**b**) fracture strain of the virtual to 0 °C shifted LME starting temperature.

Depending on the strain rate, a minimum temperature $T_{LME,\;start}$ for detectable LME can be identified. Figure 6a shows the relation between those critical temperatures and the pertaining strain rates. The data points line up on a straight line, when the abscissa values are given in a logarithmic scale. This linear dependency of $T_{LME,\;start}$ on ln$\dot{\varepsilon }$ is defined by the constants K and *p* in Equation (1). For DP1200HD, the parameters are found to be K=−26 and P=632.5. Please note that the unit system mm *n* and s is provided for all the equations in this paper, henceforth the given numbers will be valid in this unit system.

Below a specific strain rate of 5.0 × 10^−5^, no LME occurs, because diffusion processes between steel and zinc reduce the risk of embrittlement.

(1) $$T_{LME,\;start}=K\ln\left(\dot{\varepsilon }\right)+P$$

For all further considerations, the current temperature $T_{current}$ at a material point will be related to a common starting point by introducing the virtual temperature $T_{LME}$ according to Equation (2). $T_{LME}$ >0 thus signals that LME occurs. It can be seen as a first indicator for the criticality of embrittlement.

(2) $$T_{LME}=T_{current}-T_{LME,\;start}$$

As $T_{LME}$ rises, the risk of LME increases. Introducing $T_{LME}$ helps identifying a suitable description accounting for the strain rate influence in the LME damage model. The fracture strain can then be plotted as a function of $T_{LME}$ and the strain rate; see Figure 6b.

**Figure 7:**
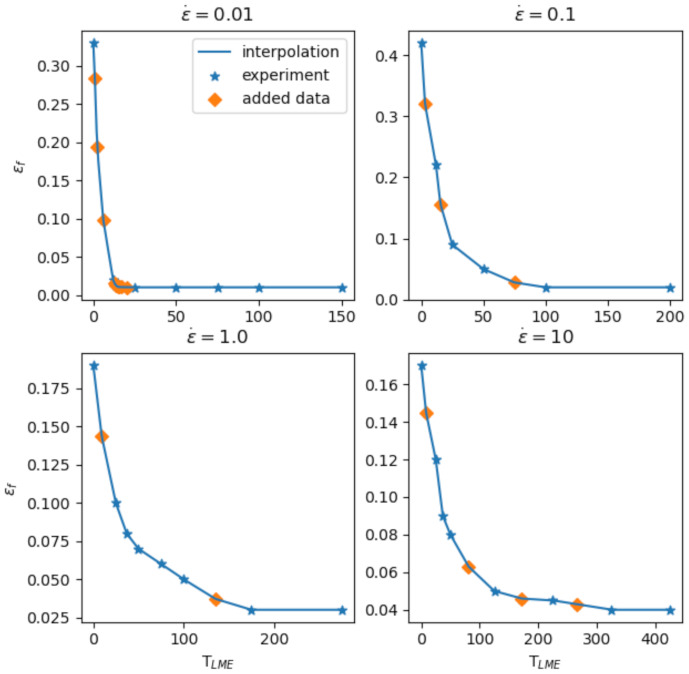
Experimental data points and added auxiliary points in temperature direction.

The experimental observations reveal that the fracture strain $\varepsilon_{f}$ depends on temperature (T), strain rate ($\dot{\varepsilon }$) and the presence of zinc ($c_{Zn}$). Clearly, without zinc, no LME would occur. Therefore, we investigated symbolic regression modeling to approximate the response surface of fracture strain only as a function of $T_{LME}$ and $\dot{\varepsilon }$. As can be seen in Figure 4, the experimental data are quite sparse, especially in the strain rate space (where data are available only for four strain rate values). Symbolic regression is a data-driven approach, where the final quality of the trained model depends on the quality and quantity of the data. The modeled response surface must respect and follow the overall trends of the data without showing any sharp or abrupt changes in temperature and strain rate direction. To mitigate the shortcomings of having few experimental data points, some auxiliary points were added along the temperature axis; see Figure 7.

Obviously, for strain rate 0.01, we have used more synthetic points to increase the robustness of the trained model and to cater for the sharply changing trend around $T_{LME}=15\;{}^{\circ}C$. After adding the auxiliary points, a 1-dimensional linear interpolation is performed to generate a denser grid in temperature direction. For the strain rate direction, since experiments are so scarce, it was more difficult to add auxiliary points in a reliable way. Therefore, we just performed 1-dimensional linear interpolation in $\dot{\varepsilon }$., considering the same temperature axis, by using the interpolated experimental data in $T_{LME}$ space. Eventually, a total of 31,050 data points was generated. Afterwards, a symbolic regression optimization is performed to find a mathematical equation which best fits the data, from a variety of functions in the search space of the genetic algorithm exponential function types combined by basic arithmetic operations which provide an optimal fit. Equation (3) shows an example of such an equation describing the inner correlations of the data satisfactorily. The model contains five parameters (*A, B, C, D, E*), each with its specific dimension. Equation (3) only works in this given unit system, while in a different unit system, the equation would have a similar structure but the exact values of *A, B, C, D* and *E* would be different. The model parameters can be readily adopted for other materials with the same method if enough experimental data is available.

(3) $$\varepsilon_{f}=\frac{A}{\frac{B-T_{LME}+\frac{T_{LME}}{\dot{\varepsilon }}}{C}+\left(\left(\;\;D+T_{LME}\;\right)-\frac{\left(E-T_{LME}\right)}{e^{\dot{\varepsilon }}}\right)}$$

$$\begin{array}{c} A=7.17\;\;\\ B=6.69\;\;\\ C=12.41\\ D=40.87\\ E=24.07 \end{array}$$

#### 3.2.2. Damage Indicator

The basic structure, as shown in Equation (4), of the damage model ($D_{LME}$) follows well-accepted ductile damage models [39,40]. The damage model can be applied for every material point in FE models and is, hence, a local model where the damage is driven by the plastic strain. In each time increment of the simulation, the plastic strain increment ($d\varepsilon_{pv}$) is related to the current fracture strain $\varepsilon_{f}$ in order to calculate the ‘consumed’ ductility of the material.

(4) $$D_{LME}=\int_{0}^{\varepsilon_{pv}}\frac{d\varepsilon_{pv}}{\varepsilon_{f}}$$

In the next step, the damage model is validated by means of a single element FE analysis that allows to numerically reproduce the hot tensile tests. The implementation of the damage model is accomplished by the user-defined subroutine UVARM in Abaqus which returns $D_{LME}$ at each integration point. The calculation starts, when the current temperature exceeds the critical temperature $T_{LME,\;start}$. Subsequently, $T_{LME}$ is calculated and with this input, the fracture strain $\varepsilon_{f}$ is found according to Equations (2) and (3). Finally, the evolution of $D_{LME}$ is calculated according to Equation (4).

**Figure 8:**
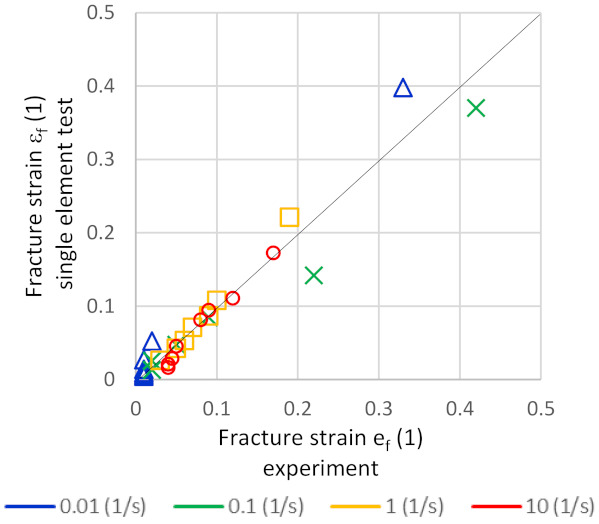
Comparison between fracture strain of the model versus experiments.

**Figure 9:**
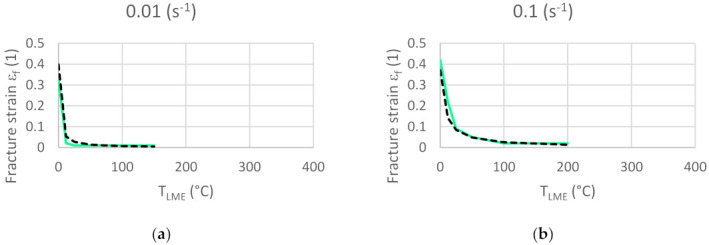

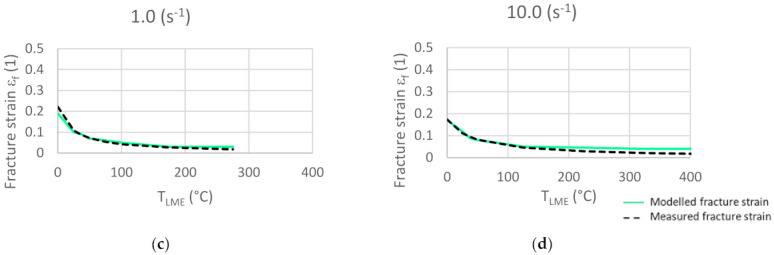
Comparison of modelled fracture strains (cyan solid line) vs. experimentally measured fracture strains (black dashed line) at strain rates of (**a**) 0.01 s^−1^; (**b**) 0.1 s^−1^; (**c**) 1.0 s^−1^ and (**d**) 10.0 s^−1^ as a function of T_LME_.

The single element test shows a good correlation between the experimental data and the model results for the fracture strain, especially for strain rates ranging from 0.01 s^−1^ up to 1.0 s^−1^; see Figure 8 and Figure 9. For RSW processes, this is the most relevant range of strain rates.

### 3.3. Model Validation

**Figure 10:**
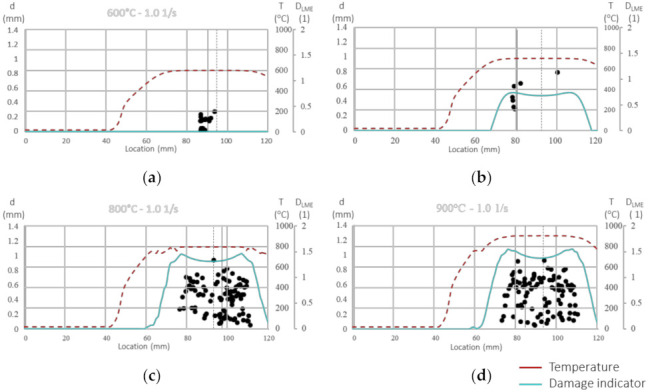
Comparison of the calculated temperature distribution T (red and dashed) and the damage indicator D (cyan) with the crack depth d measured in the samples (the path is shown in Figure 1 marked with s) in mm (black dots) and the sample center (dotted vertical line) as well as the rupture location (solid vertical line) for a strain rate of 1.0 s^−1^ at (**a**) 600 °C; (**b**) 700 °C; (**c**) 800 °C and (**d**) 900 °C.

The damage model is validated against the local microcrack networks found in a series of Gleeble^®^ experiments, including specimens with a spot weld. Figure 10 shows the distribution of the calculated damage indicator along with position and depth of micro-cracks as found in the Gleeble^®^ experiments for a strain rate of 1.0 s^−1^ and four different temperatures. The calculated temperature distribution along the center of the hot tensile specimen as indicated in Figure 1 by the variable s is displayed as dashed red line, while the damage indicator distribution is represented by the solid cyan line. A vertical dotted line marks the sample center. In the case of Figure 10a, no LME is found for a temperature of 600 °C well beyond T_LME,start_, but minor cracks with the depth d (black dots) can be found around the necking region. For 700 °C, shown in Figure 10b, deeper cracks are found, especially in the regions with a higher predicted damage indicator. Here, the final rupture takes place at the location marked by a vertical solid line which coincides well with the area that is identified as critical by the model. The region comprising LME cracks is also captured well for 800 °C, as shown in Figure 10c. For 900 °C, the position of a size of the highly cracked region is reasonably well predicted by the model, although the model shows a somewhat larger width than the experiment. Note that a value of $D_{LME}=$1 already indicates through-cracks and therefore, total rupture of the specimen. Figure 10c and d show values for $D_{LME}$ >1. This is due to the fact that the elongation in the model continues beyond the stage where, in reality, the specimen already ruptures. The fracture process itself was not modeled. Shear banding occurs due to the meshing in the middle of the model always at 90 mm. This would require element deletion techniques or a fracture mechanics analysis.

**Figure 11:**
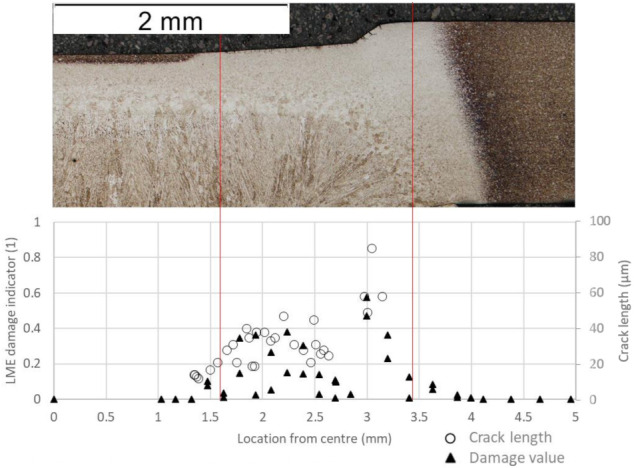
Comparison of the micrograph and the crack lengths against the damage indicator with matching high-risk zones.

After confirming the viability of the damage indicator concept, it was implemented in an FE-based RSW model. The simulations provided field quantities such as the current flow, the temperature field, local strain rates and strains necessary for computing the damage indicator in a post-processing step. For validation reasons, the simulation results showing the damage indicator is juxtaposed to the according micrograph in Figure 11. The pertaining welding conditions are described in Experimental and are electrode force = 4.5 kN, welding current = 6.9 kA, welding time = 380 ms as well as holding time = 300 ms. $D_{LME}$ stays well beyond 1 and this choice is reasonable since no through-crack is expected. The comparison of the micrograph with the values gained of the FE simulation shows a good correlation of the zones containing minor cracks (see area between vertical lines) with the surface elements exhibiting elevated values of D.

## 4. Conclusions

Hot tensile tests covering a wide temperature and strain rate range were carried out to characterize the LME behavior of a DP1200HD steel. Based on the experimental findings, a machine learning approach using symbolic regression was applied to formulate an LME damage model. The FE implementation of this model was validated against crack networks found in hot tensile tested samples as well as an exemplary resistance spot weld. The main findings of the paper are as follows:

- Hot tensile tests are an appropriate means to reproducibly and quantitively characterize the LME susceptibility of materials.
- The commonly applied rule of thumb for LME occurring in AHSS is based on plastic strain and requires temperatures to be above 700 °C. This paper offers a new perspective in that it shows that LME can be found well beneath austenite’s start temperature, which indicates that not only austenite but also ferrite and martensite are prone to LME.
- Symbolic regression was successfully applied to support the damage modeling and delivered robust results.
- The developed and FE implemented damage model was validated by two different means and can be applied in, for example, RSW process design.

## Figures and Tables

**Table 1:** Chemical composition of the DP1200HD steel.

C (%)	Si (%)	Mn (%)	Cr (%)	Cu (%)	Nb (%)	Fe
0.21	1.46	2.53	0.03	0.016	0.002	Balanced

## Data Availability

The raw/processed data required to reproduce these findings cannot be shared at this time due to technical or time limitations.
